# The Role of Optical Coherence Tomography Angiography (OCTA) in Detecting Choroidal Neovascularization in Different Stages of Best Macular Dystrophy: A Case Series

**DOI:** 10.3390/medicina57030213

**Published:** 2021-02-27

**Authors:** Yaqoob Qaseem, Olga German, Maria Vittoria Cicinelli, Rukhsana G. Mirza

**Affiliations:** 1 Department of Ophthalmology, Feinberg School of Medicine, Northwestern University, Chicago, IL 60208, USA; yaqoob.qaseem@northwestern.edu (Y.Q.); olekakh@gmail.com (O.G.); maria.cicinelli@northwestern.edu (M.V.C.); 2 School of Medicine, Vita-Salute San Raffaele University, 20132 Milan, Italy; 3 Department of Ophthalmology, IRCCS San Raffaele Scientific Institute, 20132 Milan, Italy

**Keywords:** anti-VEGF, Best disease, Best macular dystrophy, choroidal neovascularization, hereditary retinal dystrophies

## Abstract

Best macular dystrophy (BMD) is an autosomal dominant macular dystrophy of childhood onset characterized by bilateral and symmetric vitelliform lesions. Several stages of disease have been well-described in the literature. Choroidal neovascularization (CNV) has traditionally been considered a hallmark of end-stage disease, and anti-vascular endothelial growth factor (anti-VEGF) agents have been used to improve visual prognosis. While CNV was historically detected with fluorescein angiography, optical coherence tomography angiography (OCTA) has recently been employed as a novel mechanism for identifying CNV in BMD. In this case series, we discuss our institutional experience with using OCTA to detect CNV in BMD and contextualize this experience within the broader emerging literature. While OCTA allows for the identification of CNV in less severe stages of BMD, the management of this CNV remains uncertain.

## 1. Introduction

The bestrophinopathies comprise a range of hereditary retinal dystrophies resulting from pathogenic variations of the BEST1 gene, which encodes a calcium-sensitive chloride channel expressed in the retinal pigment epithelium (RPE) [1]. The most prevalent variations of this gene are inherited in an autosomal dominant manner, with one of the resulting phenotypes known as Best macular dystrophy (BMD) [1]. BMD is a slowly progressive macular dystrophy with childhood onset characterized by bilateral and symmetric vitelliform lesions in the macula [2]. Its clinical stages have been well described in the literature, and include a pre-vitelliform stage, characterized by a normal macula with abnormal electrooculogram (EOG); a vitelliform stage, characterized by “yolk-like” lesions; a “pseudohypopyon” stage with partial reabsorption of the vitelliform material; a vitelliruptive stage, which looks like a non-homogeneous “scrambled egg”; and a fifth stage featuring central RPE and retinal atrophy and a marked drop in visual acuity [3].

Choroidal neovascularization (CNV) is an uncommon complication of BMD and has traditionally been considered a hallmark of end-stage disease (stage 6); early detection and treatment with anti-vascular endothelial growth factor (anti-VEGF) agents often help to improve the visual prognosis [4,5]. Fluorescein angiography (FA) is the method of choice for diagnosing CNV; however, the diagnosis of CNV may be particularly challenging in BMD due to lipofuscin dye staining and RPE atrophic changes at the posterior pole.

Recently, optical coherence tomography angiography (OCTA) has been successfully used for detecting CNV in BMD [6]. OCTA has revealed that CNV may occur in earlier stages of the disease and might be the presenting sign in a small subset of BMD patients [7,8]. Moreover, Parodi et al. recently illustrated that the OCTA parameters of the CNV may differ between early and late stages of the disease [9]. In particular, CNV in late stages may show less exudative manifestations. In this article, we report our institutional experience with using OCTA to identify subfoveal CNV in different stages of BMD in three illustrative cases and further discuss the emerging body of literature on the topic.

## 2. Case Reports

**Figure 1:**
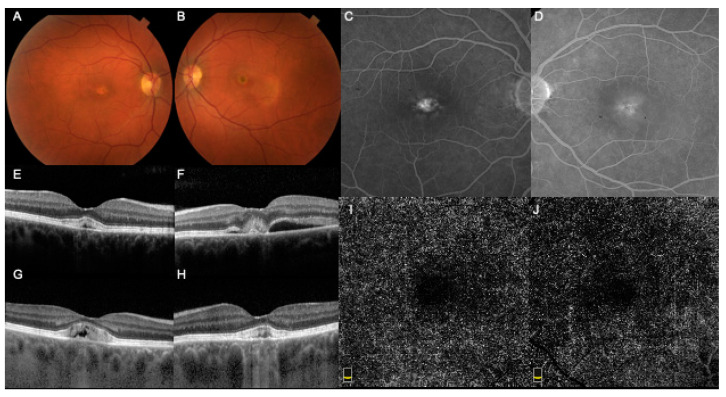
Multimodal imaging of Case 1, presenting with choroidal neovascular membrane in the left eye (LE). (**A**,**B**). Fundus Photography of both eyes showing pigmentary changes at the posterior pole in both eyes and yellowish material in the LE. (**C**,**D**). Fluorescein angiography revealing bilateral early macular hyperfluorescence, with dye leakage in the late phases in the LE. (**E**,**F**): Optical coherence tomography (OCT) disclosing subfoveal detachment filled with hyperreflective material. The LE was noticeable for retinal thickening, subretinal fluid, hyperreflective retinal pigment epithelium detachment subfoveally. (**G**,**H**). Follow-up OCT at the last visit (8 years following initial presentation) showing sustained resolution of subretinal fluid in the LE. (**I**,**J**). OCT Angiography at the last visit (8 years following initial presentation), where no clear neovascular network on outer retinal slabs was evident.

Case 1, a 47-year-old female, presented with best-corrected visual acuity (BCVA) of 20/20 in the right eye (RE) and 20/40 in the left eye (LE); she complained of recent onset of metamorphopsia in her LE. Her family history was significant for BMD in her two siblings and other members of the paternal side of the family. Anterior segment examination was unremarkable. Fundus examination showed pigmentary changes at the posterior pole in both eyes and a yellowish subretinal material inferotemporal to the macula in the LE (Figure 1A,B). The FA revealed bilateral early macular hyperfluorescence, with dye leakage in the late phases in the LE (Figure 1C,D). The spectral-domain optical coherence tomography (SD-OCT) disclosed a normal-thickness retina and subfoveal detachment filled with hyperreflective material in the RE, with scattered choroidal hypertransmission (Figure 1E). The LE was noticeable for retinal thickening, subretinal fluid, hyperreflective RPE subfoveal detachment, and hyperreflective intraretinal dots. Inferotemporally, there was subretinal fluid with hyperreflective material extending from the photoreceptors’ layer (Figure 1F). 

The patient was diagnosed with BMD complicated by CNV in the LE and underwent 4 intravitreal injections of bevacizumab, with resolution of subretinal fluid after the second injection. No recurrence of CNV activity was noted in 8 years of follow-up with SD-OCT imaging stable throughout this period. At the last visit 8 years following initial presentation, the BCVA was 20/70 in the RE and 20/50 in the LE, the SD-OCT showed a similar appearance in both eyes (Figure 1G,H), and the OCTA (which was not available 8 years earlier) did not demonstrate any clear neovascular network on outer retinal slabs (Figure 1I,J). 

**Figure 2:**
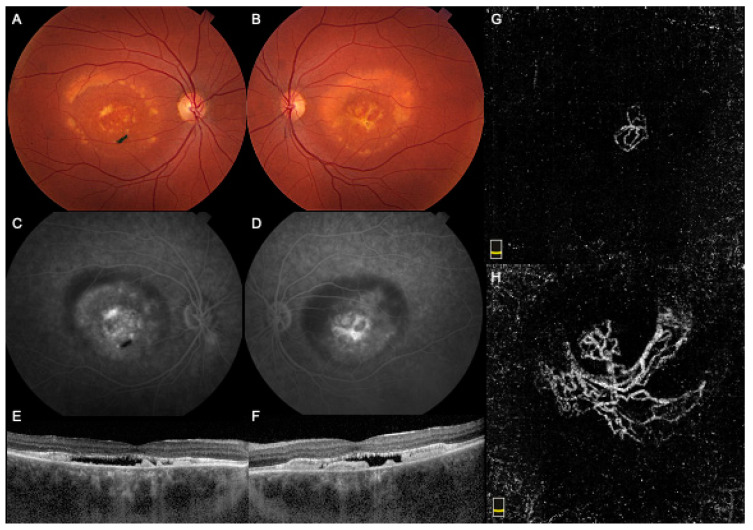
Multimodal imaging of Case 2 showing a choroidal neovascular membrane in both the right eye (RE) and left eye (LE). (**A**,**B**). Fundus color picture showing large bilateral vitelliform detachment, subretinal fibrosis, and pigmentary changes. (**C**,**D**). Fluorescein angiography showed central dye staining and late pooling within the vitelliform lesions. (**E**,**F**). Optical coherence tomography (OCT) demonstrated a shallow subfoveal optically empty space with a hyperreflective retinal pigment epithelium detachment in both eyes. (**G**,**H**). En face OCT Angiography revealed a neovascular network in the outer retinal slabs bilaterally. The right, symptomatic eye showed a smaller network with thick caliber vessels that maintained the same size despite treatment. The left, asymptomatic eye showed a large network with large caliber vessels that corresponded to the central fibrotic elements clinically and on the fundus photo.

Case 2 was a 34-year-old female who presented complaining of decreasing vision in her RE. The BCVA was 20/40 in the RE and 20/30 in the LE. Multiple family members were diagnosed with BMD, including her father. Fundus examination showed large bilateral vitelliform detachments, subretinal fibrosis, and pigmentary changes (Figure 2A,B). FA showed central dye staining and late pooling within the vitelliform lesions (Figure 2C,D). The SD-OCT demonstrated a shallow subfoveal optically empty space with a hyperreflective RPE detachment in both eyes (Figure 2E,F). B-scan OCTA showed flow pixels within the hyperreflective lesions, while the en-face slab revealed a neovascular network in the outer retinal slabs bilaterally (Figure 2G,H). The patient received two monthly injections of bevacizumab in the RE. Since there was no improvement in vision, subretinal fluid on SD-OCT, or CNV size on OCTA, further intravitreal treatment was deferred with a plan to closely observe for subjective or objective worsening. Her last BCVA was 20/70 in the RE and 20/25 in the LE, with a similar macular appearance on follow-up.

**Figure 3:**
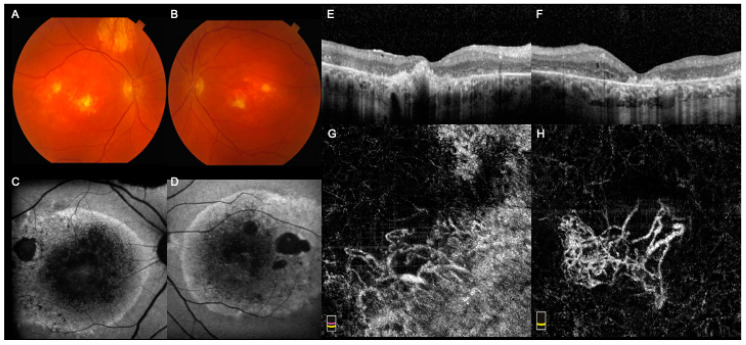
Multimodal imaging of Case 3 showing late-stage disease and choroidal neovascular membrane (CNV) in both the right eye (RE) and left eye (LE). (**A**,**B**). Fundus examination showed bilateral retinal pigment epithelium and retinal atrophy at the posterior pole, with subfoveal fibrosis in the RE and parafoveal fibrosis in the LE. (**C**,**D**). Short-wavelength fundus autofluorescence (FAF) showed a hyper-FAF ring at the posterior pole with multiple patches of hypo-FAF, corresponding to RPE atrophy. (**E**,**F**). Optical coherence tomography (OCT) revealed dense hyperreflective lesions and extensive back-scattering. (**G**,**H**). En face OCT Angiography showed a large CNV with thick caliber vessels in both eyes notwithstanding significant artifacts in the RE due to retinal atrophy. No exudation was associated with CNV in either eye.

Case 3 was a 71-year-old male presenting with BCVA of 20/150 and eccentric vision in both eyes. His ocular history was positive for EOG-proven BMD, with onset in his 5th decade of life. He had a strong family history of BMD. Fundus examination showed bilateral RPE and retinal atrophy in the posterior pole, with subfoveal fibrosis in the RE and parafoveal fibrosis in the LE (Figure 3A,B). SW-FAF showed a hyper-FAF ring at the posterior pole with multiple patches of hypo-FAF, corresponding to RPE atrophy (Figure 3C,D). SD-OCT revealed dense hyperreflective lesions and extensive back-scattering (Figure 3E,F). FA demonstrated early and late hyperfluorescence, corresponding to window defects and staining, respectively. The patient was managed conservatively and followed for 11 years. At the last visit, the patient underwent OCTA, which showed large CNV lesions in both eyes (Figure 3G,H). No exudation was associated with CNV in either eye. 

To summarize our institutional cases, we showed 5 eyes of 3 patients with BMD featuring CNV in varying stages of the disease. While two patients were in the vitelliruptive stage, one featured macular RPE and retinal atrophy, traditionally categorized as stage 5. While three eyes of two patients presented with subfoveal hyporeflectivity in the eye evidently consistent with CNV, both eyes of the third patient had subretinal fibrosis with no signs of exudation. Two eyes of two patients received anti-VEGF treatments, but only one responded successfully with both visual and morphologic improvement.

## 3. Discussion

The classification of BMD relies on fundus appearance, but the advent of SD-OCT has helped in characterizing the different presentations of the disease [10]. Traditionally, the diagnosis of CNV and macular fibrosis corresponds to the most advanced stage (i.e., stage 6) and is often accompanied by thinning of the sensory retina and diffuse loss of the RPE/photoreceptors interface [10]. The visual acuity is very poor in this stage, often below the legal threshold of blindness [11].

More recently, several studies have explored the microvascular changes occurring in BMD by means of OCTA. These studies highlighted a diffuse loss of capillary density at different depths of the retina in BMD patients [6,7]. Furthermore, OCTA has shown superior sensitivity in detecting CNV and assessing its size compared to FA, due to the masking of the CNV by staining of vitelliform lesions seen in FA [12]. Interestingly, these studies reported a far greater prevalence of CNV (up to 39%) compared with prior studies based on traditional imaging, which instead suggested a prevalence of CNV in BMD of 2-9% [6,12]. One further reported that 21 of 24 (88%) eyes with CNV were in either the vitelliruptive or atrophic stage, which was consistent with our institutional experience [6].

Case 2 and 3 in our cohort support the increased sensitivity of OCTA in detecting CNV that may not be definitively visualized on FA. The ability to recognize CNV with OCTA in Best disease leads us to believe that CNV is more prevalent in BMD than originally reported and may occur earlier in the disease process. In this view, a re-classification of the BMD stages, based on multimodal imaging and OCTA findings, might be warranted. 

One recent study by Parodi et al. suggests that subfoveal CNV identified by OCTA in the vitelliform and pseudohypopyon stages may be distinct from that of the vitelliruptive and atrophic stages. In their study, only eyes in the vitelliform and pseudohypopyon stages showed exudative changes, and treatment with CNV led to stabilization of lesion size with improvement in visual acuity [9]. Contrastingly, at our institution, Case 1 and Case 2 both described patients with exudative changes secondary to CNV in the vitelliruptive stage of disease. In Case 1, anti-angiogenic treatment led to improvement in macular morphology and stabilization of visual function, with sustained results on long-term follow-up. By contrast, Case 2 showed a poor response to anti-VEGF treatment, with no changes in the subretinal hyporeflectivity. In these cases, the SD-OCT findings should be interpreted either as optically empty spaces resulting from lipofuscin reabsorption or as subretinal fluid secondary to dysfunctional RPE pump, rather than signs of CNV exudation [13]. Finally, Case 3 described a patient presenting with an incidental finding of bilateral inactive CNV in the setting of advanced atrophic disease. This was more consistent with the late-stage CNV reported by Parodi et al. Since most of the descriptions of CNV in BMD belong to sporadic case reports or short case series, tailoring treatment plans in this disease remains a challenge.

Furthermore, recent literature by Murro et al. has shown that treatment of CNV in BMD leads to a remodeling and an initial reduction of the CNV on OCTA, consistent with the conclusions in our case series [14]. We believe that this remodeling could represent an advantageous process in this disease; given this emerging evidence, OCTA’s role in identifying CNV in BMD may be of even greater importance.

Based upon the identification of two distinct types of CNV in early and late stages of disease, Parodi et al. postulated differing mechanisms of neovascularization depending upon the stage of the disease [9]. While the occurrence of CNV in BMD has been associated with extensive outer retinal damage, the finding of choroidal neovascular networks in less severe stages may support a different mechanism in its pathogenesis. Nevertheless, the events inducing CNV formation and its role in disease progression remain unknown. 

Alongside the potential benefits of OCTA, this modality has several limitations. OCTA does not show leakage associated with the CNV in contrast to traditional dye angiography. This is a benefit in that it allows clearer visualization of the CNVM; however, this also does not allow visualization of the exudative activity of the neovascular network and might not represent a useful endpoint in the assessment of the therapeutic response if there is no change in the lesion size. Another limitation of OCTA is the presence of segmentation artifacts, which might be more evident in patients with severely disrupted macular anatomy, such as inherited macular dystrophy patients. Finally, projection artifacts from the overlying retinal vessels to the surface of the vitelliform subretinal material may be a source of pseudoflow signal and might be misinterpreted as CNV [15].

## 4. Conclusions

In summary, our case series of this relatively rare condition adds to the literature illustrating the emerging role of OCTA in the early diagnosis of CNV complicating BMD. OCTA has been shown to demonstrate a greater prevalence of CNV in Best disease than previously thought and may allow for its detection even in the absence of clear signs suggestive of CNV on traditional tests. Furthermore, OCTA has been used to identify CNV in less severe stages of the disease, sometimes before complete reabsorption of the vitelliform material and development of macular atrophy. Further research is needed to fully elucidate the mechanism and role of CNV occurring in BMD and the utility of multimodal imaging in the therapeutic management of the disease.

## Data Availability

The data presented in this study are available on request from the corresponding author. The data are not publicly available due to privacy restrictions.
